# Seismic Performance of Beam–Column Joints in Seawater Sand Concrete Reinforced with Steel-FRP Composite Bars

**DOI:** 10.3390/ma18102282

**Published:** 2025-05-14

**Authors:** Ruiqing Liang, Botao Zhang, Zhensheng Liang, Xiemi Li, Shuhua Xiao

**Affiliations:** 1 Guangzhou Electric Power Design Institute Co., Ltd., Guangzhou 510060, China; rq_liang0303@163.com (R.L.); botaoz2024@163.com (B.Z.); zhensheng_l@163.com (Z.L.); 2 School of Civil and Transportation Engineering, Guangdong University of Technology, Guangzhou 510006, China; 18819487074@163.com; 3 School of Environment and Civil Engineering, Dongguan University of Technology, Dongguan 523080, China

**Keywords:** Steel-FRP composite bar (SFCB), beam–column joints, seawater sand concrete, seismic performance, energy consumption

## Abstract

Steel fiber-reinforced polymer (FRP) composite bars (SFCBs) combine the ductility of steel reinforcement with the corrosion resistance and high strength of FRP, providing stable secondary stiffness that enhances the seismic resistance and safety of seawater sea–sand concrete structures. However, the seismic performance of SFCB-reinforced seawater sea–sand concrete beam–column joints remains underexplored. This study presents pseudo-static tests on SFCB-reinforced beam–column joints with varying column SFCB longitudinal reinforcement fiber volume ratios (64%, 75%, and 84%), beam reinforcement fiber volume ratios (60.9%, 75%, and 86%), and axial compression ratios (0.1 and 0.2). The results indicate that increasing the axial compression ratio enhances nodal shear capacity and bond strength, limits slip, and reduces crack propagation, but also accelerates bearing capacity degradation. Higher column reinforcement fiber volumes improve crack distribution and ductility, while beam reinforcement volume significantly affects energy dissipation and crack distribution, with moderate volumes (e.g., 75%) yielding optimal seismic performance. These findings provide insights for the seismic design of SFCB-composite-reinforced concrete structures in marine environments.

## 1. Introduction

With economic growth and the rising demand for construction, concrete usage has expanded significantly. However, extensive use of river sand negatively impacts river ecosystems, navigation, and flood control [1,2,3]. Sea sand, preferred for its low mud content, good grading, and excellent workability [4,5,6], shares a similar mineral composition with river sand, and seawater sea–sand concrete generally performs comparably to ordinary concrete, meeting engineering requirements [7]. Nevertheless, the chloride ions in seawater sand accelerate corrosion in steel reinforcement, which can compromise concrete structures, reduce their service life, and even cause the phenomenon of “seawater sand house” [8], limiting seawater sand concrete applications. Various corrosion mitigation methods, such as galvanizing, stainless steel, and epoxy coatings, have been tested but remain suboptimal [9]. Fiber-reinforced polymer (FRP) reinforcement, known for its strength and corrosion resistance, has gained attention as an alternative to steel in concrete structures [10]. However, pure FRP reinforcement increases brittleness and reduces deformation capacity, resulting in structural responses unlike conventional reinforced concrete (RC) [11]. To address these limitations, researchers have proposed steel-FRP composite bars (SFCB), which combine the strengths of steel and FRP, offering excellent mechanical properties with stable post-yield stiffness and high ductility [12,13,14,15].

SFCB combines the corrosion resistance of FRP reinforcement with the ductility and stable post-yield stiffness of steel [16], while also exhibiting excellent short- and long-term bonding with concrete [12,17,18], thereby enhancing overall structural performance. Dong et al. [19] found that the maximum bond strength between SFCB and sea–sand concrete increased by 14.9%, 17.4%, and 7.1% after 30, 60, and 90 days of seawater dry–wet cycling, respectively, supporting its potential for seawater sea–sand concrete applications [20]. In additional studies, Xiao et al. [21] reported that SFCB beams demonstrate strong ductility, with load-deflection curves that harden post-yield, and increase in ultimate load, bending moment, ductility, and energy absorption with thicker GFRP protective layers. Ge et al. [22] found that as the area ratio of SFCB inner core reinforcement increased, the deflection and crack width of RC columns decreased, while the ultimate load rose due to higher core reinforcement yield strength. Han et al. [23,24] observed that SFCB beams designed for equal stiffness showed similar damage modes to ordinary reinforced beams, with comparable deflections and controlled crack widths. However, they demonstrated a 14.3–54.5% higher load capacity and 47.0–189.1% increase in ductility coefficient. Ding et al. [25] highlighted the superior stiffness, load capacity, and crack control of SFCB-RC members compared to standard RC. Similarly, quasi-static tests by Sun et al. [26] on SFCB-reinforced precast concrete columns showed smaller residual drifts at equivalent lateral displacements. Su et al. [27] reported that, under low circumferential cyclic loading, SFCB beams had comparable cumulative energy consumption to conventional RC beams but showed enhanced seismic performance due to concrete stabilization restraints, achieving a 27.0% increase in load capacity and 36.5% in energy consumption. Additionally, Su et al. [28] developed an analytical model for long-term seismic performance based on bond strength degradation, confirming its accuracy experimentally. This model showed that the seismic performance of SFCB-RC beams in marine environments significantly degrades within the first year, stabilizing after three years of corrosion. Consequently, SFCB is regarded as a promising alternative to steel reinforcement in marine and related structural applications.

Special attention must be given to seismic effects when applying SFCBs, as maintaining the integrity of beam–column joints during earthquakes is crucial. Ensuring that joints exhibit ductile failure modes enhances structural seismic performance [29,30,31]. Extensive research on reinforced concrete frame joints over recent decades has led to theoretical models, computational methods, and design codes worldwide, promoting safety and reliability in structural design [32,33]. Various shear load capacity models have been developed, including shear friction, empirical equations, constraint, combined block, diagonal compression bar, and truss models [34]. A variety of studies have investigated the use of FRP for strengthening existing beam–column joints to enhance seismic performance. Typically, carbon fiber, glass fiber, or basalt fiber reinforced polymer sheets are applied circumferentially around the joint core to confine concrete deformation, thereby significantly improving the shear capacity and ductility of the joint [35,36,37]. However, the inherently brittle nature of FRP materials often leads to sudden interfacial debonding failures [38]. In comparison, the seismic performance of SFCB-reinforced joints is less studied. Xu [39] found that SFCB concrete joints with basalt or glass fiber wraps demonstrated superior seismic performance, such as improved energy consumption and ductility, maintaining high load capacity post-yield and exhibiting good secondary stiffness. Conversely, SFCB joints wrapped with carbon fibers had reduced ductility, which is less favorable for seismic resistance. Currently, research on the seismic behavior of SFCB-reinforced concrete joints remains limited, especially in fully replacing steel reinforcement. Key aspects such as force-transfer mechanisms, damage modes, energy consumption, ductility, stiffness, and stress–strain behavior of SFCB-reinforced seawater sea–sand concrete joints are not well understood, restricting the practical application of SFCBs. Thus, further study on the seismic performance of SFCB frame joints is essential.

In this paper, a test program involving six SFCB-reinforced seawater sea–sand concrete beam–column joint specimens was designed and implemented to assess the seismic performance of these new joints. The test parameters included axial compression ratio, column SFCB longitudinal reinforcement fiber volume rate, and beam SFCB longitudinal reinforcement fiber volume rate. The results are presented and discussed concerning damage modes, hysteresis curves, skeleton curves, energy consumption analysis, and beam–column end angles. This study aims to enhance the seismic performance data of SFCB-reinforced seawater sea–sand concrete structures and establish a solid foundation for the future application of SFCBs. This study systematically investigates the effects of SFCB parameters (e.g., fiber volume ratio, axial compression ratio) on the seismic performance of beam–column joints, aiming to address these gaps and provide design recommendations for marine applications.

## 2. Experimental Programs

### 2.1. Seawater Sea–Sand Concrete

In this study, C30 seawater sea–sand concrete was formulated with the following proportions: cement: sand: stone: fly ash: mineral powder: water: water reducer = 1:3.08:4.09:0.215:0.215:0.67:0.031, resulting in a water–binder ratio of 0.47 and a sand rate of 43%. The specific dosages are detailed in Table 1. Ordinary silicate cement with a strength grade of 42.5 R was used. The sea sand, sourced from Zhanjiang, falls within grading area II, with a fineness modulus of 2.8, bulk density of 1520 kg/m^3^, and apparent density of 2630 kg/m^3^. Crushed stone was obtained from Qingyuan, and its performance indexes are shown in Table 2. The fly ash used was Grade II, with a mixing amount of 15%, a loss on ignition of 2.16%, a moisture content of 0.1%, and a water demand ratio of 101%. The mineral powder was S95 grade, with a dosage of 15%. A polycarboxylic acid water-reducing agent was employed at 2.2%, achieving a water reduction rate of 25.4%, a water ratio of 58%, a solids content of 10.88%, a density of 1.025 kg/cm^3^, and a chloride ion content of 0.02%. Additionally, artificial seawater, formulated according to ASTM D1141-98 [40], was used, with the specific configuration ratio detailed in Table 3.

The mechanical properties of the concrete were tested according to GB/T 50081-2019 [41] and ASTM C469 [42]. Three 150 mm cube samples and three cylinders were prepared for curing, followed by compressive testing using an MATEST-500t universal testing machine. The cubic and cylindrical compressive strengths of the concrete were found to be 32.59 MPa and 29.42 MPa, respectively, with a modulus of elasticity of 27.73 GPa.

### 2.2. Mechanical Properties of SFCBs

**Figure 1:**
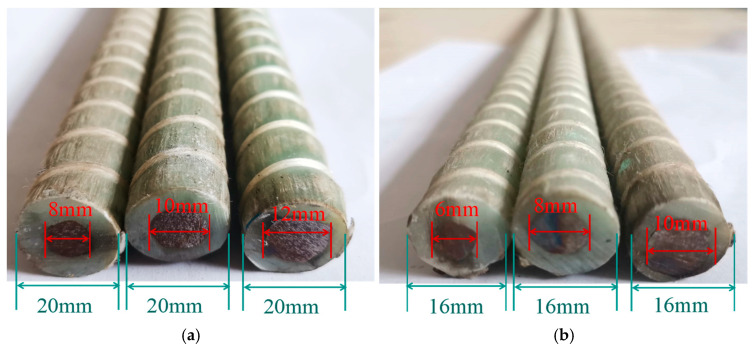
Longitudinal bars: (**a**) column longitudinal bars; (**b**) beam longitudinal bars.

**Figure 2:**
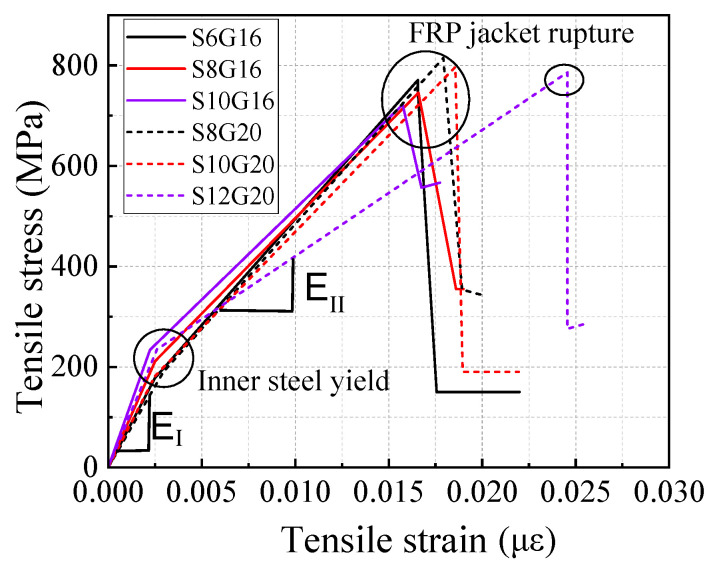
SFCB stress–strain curves.

**Figure 3:**
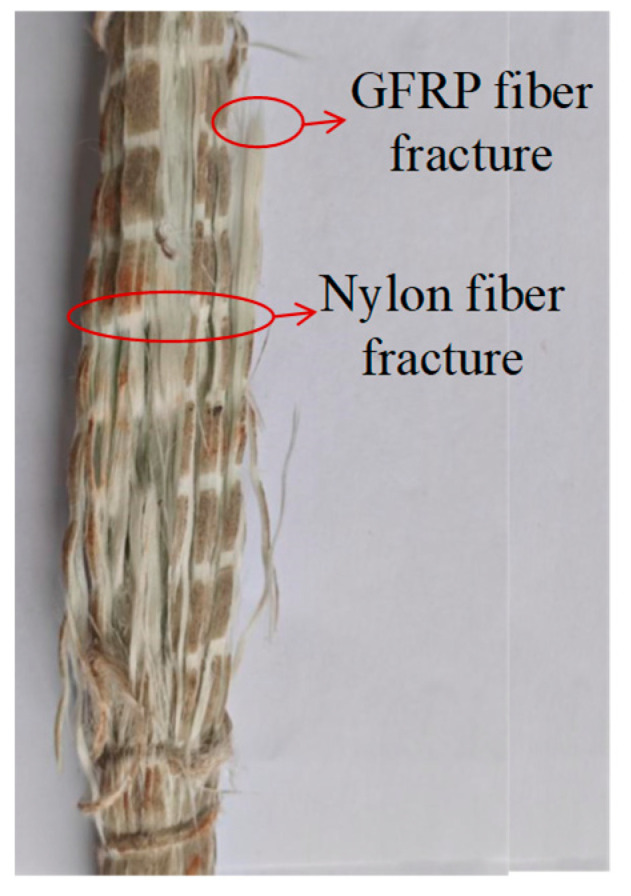
SFCB failure mode.

The SFCB consists of an HRB400 steel core and an outer layer of GFRP. The HRB400 steel bars are available in diameters of 8 mm, 10 mm, and 12 mm, with the outer fiber layers having thicknesses of 6 mm, 5 mm, and 4 mm, respectively, as shown in Figure 1. All bars were produced or supplied by Shenzhen Haichuan New Materials Technology Co., Ltd. (Shenzhen, China). The fibers for the composite bars and GFRP bars are made from glass fibers produced by Chongqing International Composite Materials Co., Ltd. (Chongqing, China). The resin matrix used is unsaturated polyester resin. The composite bars are manufactured using pultrusion technology and reinforced with nylon fibers. Following the GB/T 30022-2013 [43], tensile tests were conducted on SFCBs. Seamless steel tubes served as anchors at both ends of the tendons, with expanded cement used as the filler material. Five identical specimens for each reinforcement material were prepared, and the sampling lengths are presented in Table 4. The tensile tests were performed using a material testing machine with a loading speed of 2 mm/min. The basic mechanical properties of the SFCB core reinforcement were evaluated in accordance with the GB/T 228.1-2010 [44]. Additionally, the load was measured by the force sensor of the testing machine, while the strain was measured using an extensometer and strain gauges at the center of the specimen. The extensometer is integrated with the tensile testing machine, and the strain gauges are supplied by Chengdu Siweide Technology Co., Ltd. (Chengdu, China). The load and strain data were collected using a TDS-530 static data acquisition system supplied by Tokyo Measuring Instruments Lab. (Tokyo, Japan). The mechanical properties of the bars are summarized in Table 5 and illustrated in Figure 2. When the inner-core steel bars yield, the yield strength of the SFCB is calculated by dividing the tensile force by the area of the composite bar, and therefore it is lower than the yield strength of the steel bars. The stress–strain curves of the SFCB exhibited distinct bipartite stiffness, and the damage mode was characterized by typical necking of the inner-core reinforcement following the failure of the outer fiber layer (Figure 3).

### 2.3. SFCB Reinforced Concrete Beam–Column Joints Tested

In this study, pseudo-static tests were conducted on column joints in SFCB-reinforced seawater sea–sand concrete structures. In frame structures, there is no bending moment at the counterbending points of beams and columns, and no vertical uniform load is applied to the beams during testing. Therefore, the section between the counterbending points of the beams and columns was selected as the test object to accurately reflect the structural response [45]. Following the GB 50011-2010 [46], the joints were designed according to the principle of “strong joints and weak members, strong columns and weak beams”, resulting in three groups of six joint specimens. The longitudinal reinforcement of the beams consists of steel-FRP composite reinforcement with a diameter of 16 mm, while the column longitudinal reinforcement uses steel-FRP composite reinforcement with a diameter of 20 mm. The hoop reinforcement comprises glass fiber reinforcing bars with a diameter of 10 mm, spaced at 100 mm. The variables in this study include the axial compression ratio and the fiber volumetric rates of the longitudinal reinforcement for both the column and beam SFCB. The design parameters of the specimens are presented in Table 6. The concrete cover of the beam is 25 mm, and that of the column is 30 mm.

**Figure 4:**
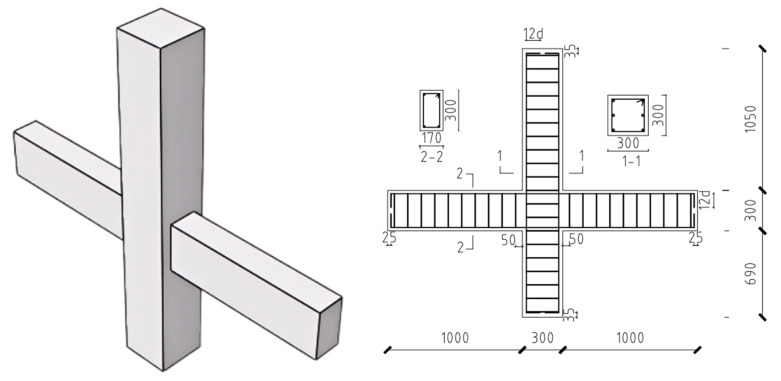
Joint size.

As shown in Figure 4, The dimensions of the column cross-section are as follows: the total height is 2040 mm, with the upper column measuring 1050 mm and the lower column 690 mm. The column is reinforced with six 20-mm SFCB longitudinal bars. Additionally, the hoops are configured with FRP reinforcement of Φ10@100 along the full length. The beam cross-section has a span of 1000 mm on both sides. It includes four 16-mm SFCB longitudinal bars at the upper and lower corners, with inner core diameters of 6 mm, 8 mm, and 10 mm made from HRB400 rebar, and outer fiber thicknesses of 5 mm, 4 mm, and 3 mm for the SFCB. The hoop bars are also made of FRP and are arranged continuously along the full span. The dimensions and reinforcement details of the joint are shown in Figure 3. The specimens are named based on the core steel of the SFCB in the longitudinal reinforcement. The letter “C” indicates column longitudinal reinforcement, while “B” indicates beam longitudinal reinforcement, with the number following each letter representing the core steel diameter of the SFCB. For example, C8B10-0.2 refers to a nodal specimen with SFCB core reinforcements of 8 mm in the column and 10 mm in the beam longitudinal bars, respectively, and an axial compression ratio of 0.2. The axial compression ratio refers to the ratio of the applied axial force to the product of the concrete strength and the cross-sectional area.

### 2.4. Loading and Measurement Programs

**Figure 5:**
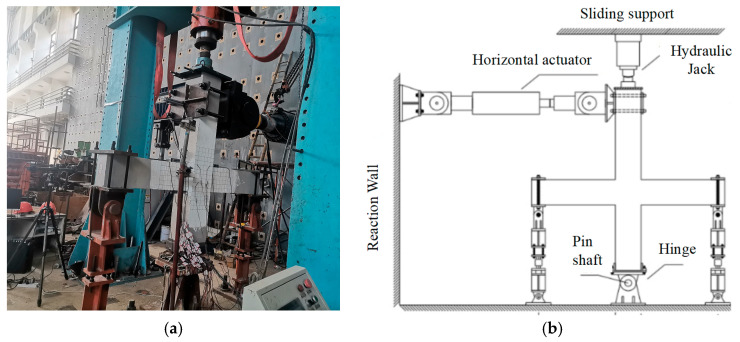
Test set-up: (**a**) joint specimen; (**b**) schematic drawing.

**Figure 6:**
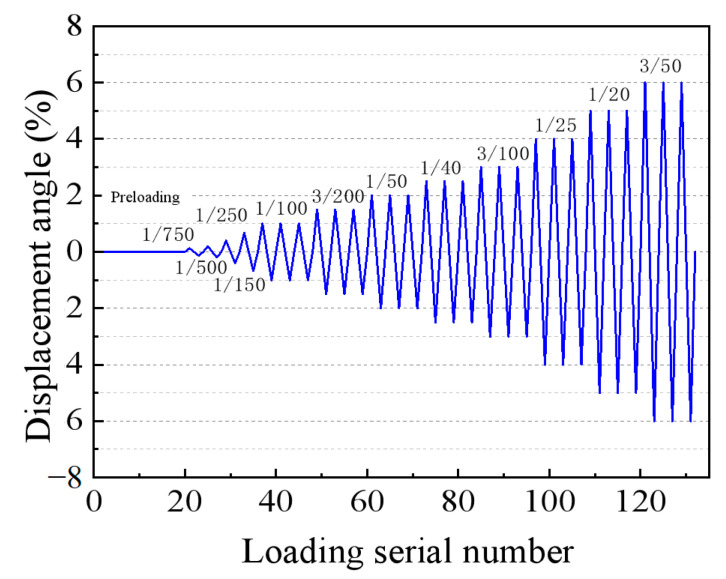
The loading scheme of the specimens.

A 500-ton hydraulic jack was connected to the upper column to apply axial force, while a 300-ton Zhoubang actuator, also connected to the upper column, was used to apply low-cycle horizontal loading. Data acquisition was conducted using a TZT3826E static collector, and the loading equipment is illustrated in Figure 5. The tests adhered to the specifications outlined in JGJ/T101-2015 [47]. The loading procedure included two preloads, each not exceeding 30% of the cracking load, followed by sequential application of 40% and 60% of the cracking loads, gradually increasing to 100% of the cracking load. The load was repeated once for each level of displacement angle before yielding of the specimen, and three times for each level of interstory displacement angle after yielding, until the bearing capacity dropped to 85% of the peak value or severe damage occurred. The test utilized column end loading with displacement control, and the specific loading steps are detailed in Figure 6. The loading process was divided into several stages, with sequential loads applied to 1/750 (0.13%), 1/500 (0.20%), 1/250 (0.40%), and 1/150 (0.67%) of the interlayer displacement angle, with only one application at each of these levels. Subsequent loading involved reaching 1/100 (1%), 3/200 (1.5%), 1/50 (2%), 1/40 (2.5%), 3/100 (3%), 1/25 (4%), 1/20 (5%), and 3/50 (6%) of the interlayer displacement angle, with three repetitions for each layer. The drift angle shown in Figure 6 refers to the interstory drift angle, and the loading sequence indicates the N-th cycle of loading.

**Figure 7:**
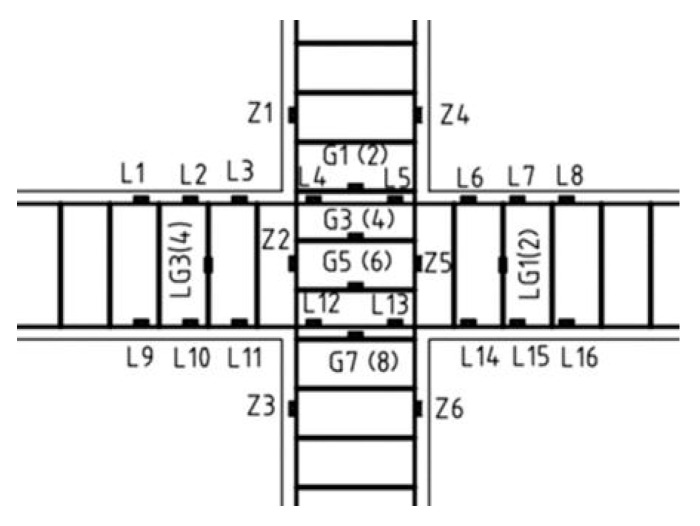
Reinforcement strain gauge layout.

**Figure 8:**
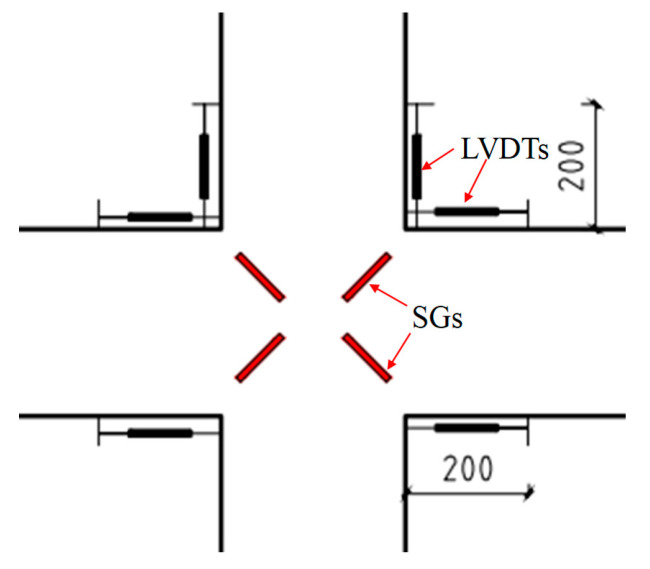
LVDTs and joint core area strain gauge arrangement.

In this test, the force-displacement data was collected using an electro-hydraulic servo system. To monitor the strain and deformation behavior in critical areas, strain gauges (SGs) and linear variable displacement transducers (LVDTs) were strategically placed on the longitudinal and hoop reinforcement, as well as in the core area of the joint. During the test, displacement and strain data were collected using the TZT3826E static data acquisition system. Specifically, the longitudinal reinforcement of the beam was equipped with 20 mm long strain gauges, each 300 mm apart. For the longitudinal reinforcement of the column, one strain gauge was positioned 150 mm above and below the core area. The hoop reinforcement gauges were placed near the core area to capture relevant data. To measure the corners of the beam and column ends, four displacement gauges were installed on the top and bottom of the left and right beams, each positioned 200 mm from the column face. On the left and right columns, two displacement gauges were arranged on each side at a distance of 200 mm from the beam face to measure the column end corners. Additionally, four strain gauges were affixed at the corners of the concrete surface in the core area of the joint to monitor deformation during the test. The arrangements of these gauges are illustrated in Figure 7 and Figure 8.

## 3. Results and Discussion

### 3.1. Failure Modes

**Figure 9:**
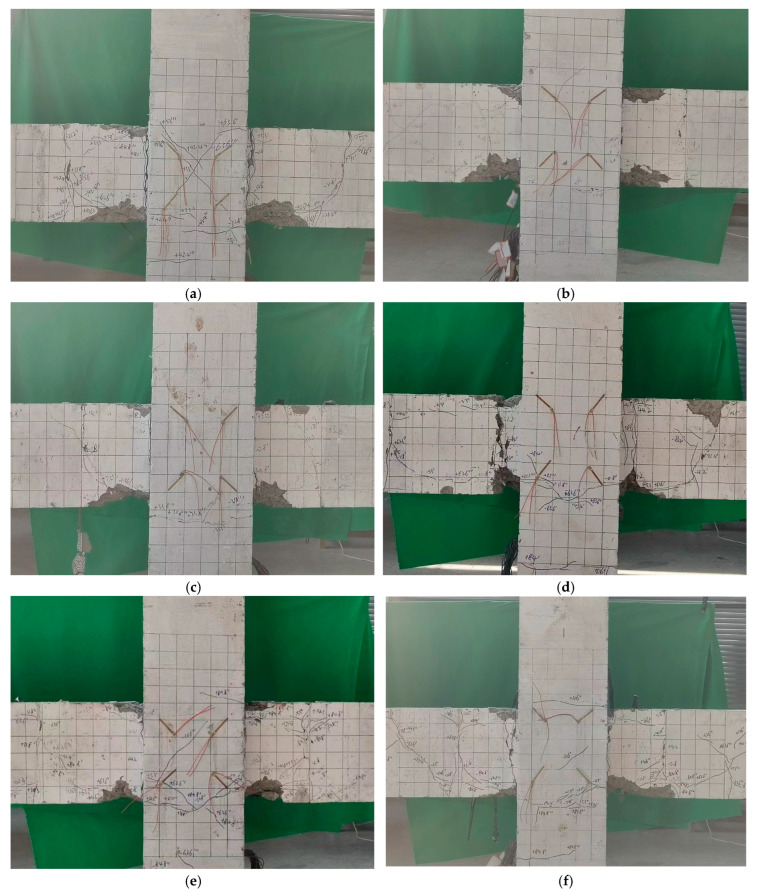
Failure modes: (**a**) C10B10; (**b**) C8B10-0.2; (**c**) C12B10; (**d**) C8B6; (**e**) C8B8; (**f**) C8B10.

As seen in Figure 9, in specimen C8B10, initial cracks appeared in the right beam–column contact section at an interstory displacement angle of 1/750. By the time the displacement angle reached 1/500, cracks had also formed in the left beam–column contact section. As the displacement angle increased to 1/100 and 3/200, crack propagation intensified, resulting in additional cracks appearing in the beams. At an interstory displacement angle of 1/40, diagonal cracks emerged in the core region of the joint, forming cross-cracks. Ultimately, when the displacement angle reached 1/20, a horizontal crack in the lower part of the left beam extended 200 mm, leading to a penetrating crack in the contact section between the beam and column, concrete spalling, and the termination of the test. Throughout the loading process, the FRP reinforcement in the specimen exhibited no slip damage, indicating a strong bond.

The damage patterns of the other specimens were similar to that of C8B10, all displaying bending failure at the beam ends. Unlike FRP-strengthened joints where debonding dominates [35], no interfacial slip occurred in SFCB specimens, validating their superior bond durability in seawater environments [18]. This behavior aligns with Dong et al.’s [19] findings on SFCB-concrete bond enhancement under cyclic corrosion. However, there are differences in the crack development and damage progression of the specimens. For instance, specimen C8B10-0.2 had fewer cracks, which were primarily concentrated at the beam end, with large cracks extending from initial fractures. The cracks in the joint core were finer, and the concrete spalling was significant. This behavior can be attributed to the increased axial compression ratio, which enhances the nodal shear bearing capacity and the bond strength of the beam’s longitudinal tendons in the core region, thereby reducing slip and effectively limiting crack propagation in the joint core [48]. When comparing specimens C8B10, C10B10, and C12B10, C8B10 exhibited the most extensive and widely distributed cracks at the beam end, along with more cracks in the lower column section. Specimen C10B10 followed with a moderate number of cracks, while C12B10 showed the least amount of cracking. This variation may be linked to the stiffness of the SFCB in the columns: the lower stiffness in column C8B10 allowed the beam to better absorb horizontal loads, leading to more fully developed cracks. Conversely, the greater stiffness of the SFCB longitudinal reinforcement in the columns of C12B10 increased the joint stiffness and correspondingly reduced beam cracking. In another comparison involving C8B6, C8B8, and C8B10, C8B6 demonstrated the least number of cracks, which were wider and accompanied by severe concrete spalling. C8B8 had a greater number of finer cracks and exhibited better concrete integrity, while C8B10 had both a larger number of cracks and a wide distribution. The stabilizing secondary stiffness of the SFCB contributed to crack extension while effectively confining the concrete. However, the lower stiffness of the longitudinal bars in the SFCB beams of C8B6 resulted in excessive concrete cracking under similar displacement conditions, leading to more severe concrete damage and overall specimen deterioration.

### 3.2. Hysteresis Curves

**Figure 10:**
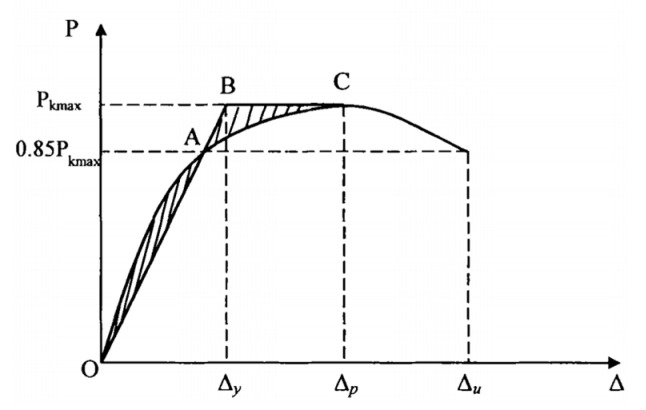
Yield point determination method.

Figure 10 illustrates the hysteresis curves of lateral load versus column drift ratio at the column loading point for each specimen. Under cyclic loading, the horizontal load–displacement curve at the top of the column forms multiple loops, known as hysteresis loops. These loops reflect the deformation characteristics, energy dissipation capacity, and stiffness degradation of the structure during cyclic loading, and are important indicators of seismic performance [28,45]. In order to accurately determine the yield displacement of the joint, the energy equivalence method is used, and the specific procedure is shown in Figure 10. First, a point A is identified on the skeleton curve. Then, a polyline OA-BC is drawn such that the area enclosed by this polyline and the X-axis is equal to the area enclosed by the skeleton curve OAC and the X-axis. In other words, the two shaded areas in Figure 10 are equal. When these areas are equal, the displacement value corresponding to point B on the polyline OA-BC, along the horizontal axis, is taken as the yield displacement of the joint. The ductility factor of the specimen is shown in Table 7. Table 7 summarizes the key results, including yield load (P*_y_*), yield displacement (Δ*_y_*), peak load (P*_m_*), peak displacement (Δ*_m_*), ultimate load (Pu), ultimate displacement (Δ*_u_*), and displacement coefficient (DC). The ductile properties of the specimens are characterized using the displacement ductility coefficient, defined as the ratio of the ultimate displacement to the yield displacement of the specimen (Δ*_u_*/Δ*_y_*).

**Figure 11:**
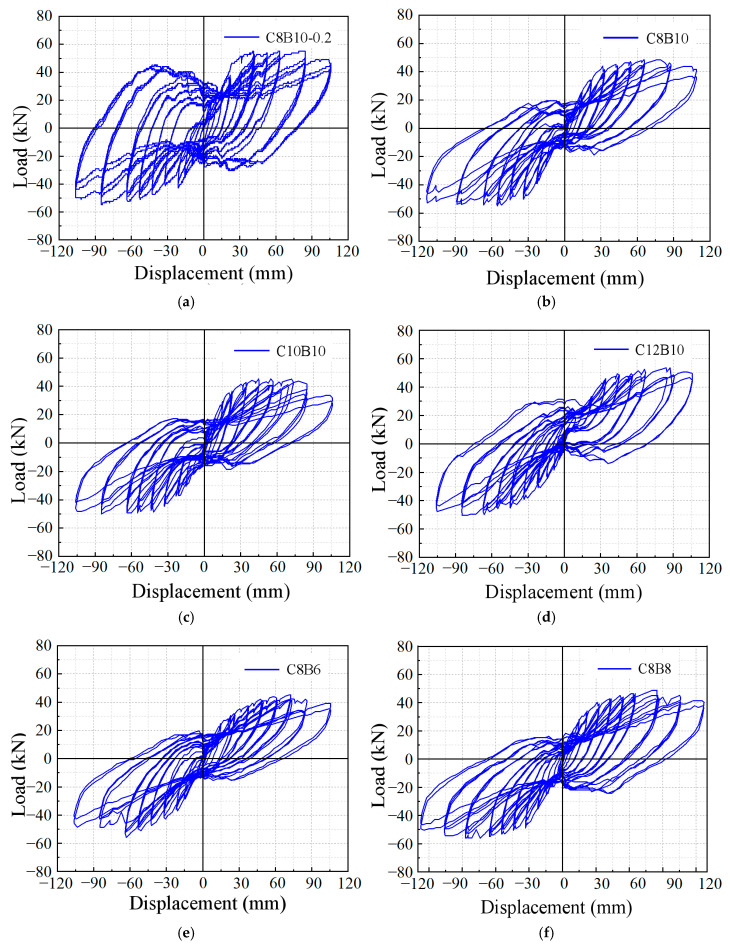
Hysteresis curves of specimens: (**a**) C8B10-0.2; (**b**) C8B10; (**c**) C10B10; (**d**) C12B10; (**e**) C8B6; (**f**) C8B8.

The hysteresis curves of specimens C8B10-0.2 and C8B10 (Figure 11a,b) exhibit an inverse S-shape, demonstrating a “pinching” effect. In the initial elastic stage, the hysteresis curves of both specimens largely overlap, with no significant residual deformation. As the interlayer displacement angle increases, the specimens enter the yielding stage, leading to residual deformation upon unloading. Stiffness gradually decreases, and the growth rate of the hysteresis curve area slows, indicating reduced energy-dissipating capacity. Eventually, the bearing capacity of the specimens diminishes, and the pinch effect becomes pronounced. Notably, C8B10-0.2 shows a higher load-carrying capacity and a fuller hysteresis loop compared to C8B10, consistent with Su et al.’s [27] observation that higher axial compression ratios enhance energy dissipation in SFCB-reinforced beams. However, this improvement is accompanied by accelerated stiffness degradation, suggesting a trade-off between strength and ductility in high-axial-load scenarios.

The hysteresis curves of specimens C8B10, C10B10, and C12B10 (Figure 11b–d) also follow an inverse S-shape, with minimal differences in the area of the single-cycle hysteresis loops. Their residual deformations are comparable, indicating that the fiber volume ratio of the longitudinal reinforcement in the column SFCB has a limited impact on the hysteresis curves. However, the hysteresis area and load capacity of C12B10 are slightly higher, showing increases of 8% and 16.7% compared to C8B10 and C10B10, respectively. This improvement may be attributed to the greater stiffness of the column longitudinal reinforcement in C12B10, which enhances lateral stiffness. In contrast, the hysteresis curves of specimens C8B6, C8B8, and C8B10 (Figure 11b,e,f) demonstrate a significant influence of the fiber volume ratio of the longitudinal reinforcement in the beam SFCB on seismic performance. In this group, C8B8 and C8B10 exhibit similar maximum load-carrying capacities and single-cycle hysteresis loop areas, whereas C8B6 shows lower energy consumption capacity and load-carrying capacity. The 4.8% increase in load-carrying capacity of C8B8 over C8B6 may be attributed to the higher fiber volumetric rate, which enhances secondary stiffness and improves the seismic energy consumption capacity of the joints. Conversely, the smaller diameter of the inner core reinforcement in C8B6 resulted in insufficient stiffness of the SFCB, hindering effective crack control and leading to severe concrete damage and relatively poor seismic performance.

### 3.3. Skeleton Curves

**Figure 12:**
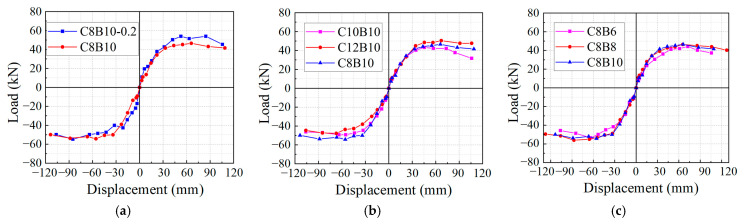
Skeleton curves of specimens: (**a**) C8B10-0.2 and C8B10; (**b**) C12B10, Z10L10, and C8B10; (**c**) C8B6, C8B8, and C8B10.

The skeleton curve represents the envelope created by connecting the maximum horizontal loads of the specimens under identical loading displacements, reflecting the various stages and characteristics of force and deformation in the members. As shown in Figure 12a, the skeleton curves of specimens C8B10-0.2 and C8B10 initially coincide, presenting a linear form. This indicates that the axial compression ratio does not significantly affect the bearing capacity and stiffness of the specimens during the early loading stage, where the joint remains in the elastic phase. As the interlayer displacement increases, the joint experiences plastic deformation, causing the curve’s slope to gradually decrease, which reflects a reduction in joint stiffness. In contrast, the bearing capacity of C8B10-0.2 increased by 16%, but the rate of decline was more rapid. This suggests that while a higher axial compression ratio enhances stiffness and bearing capacity, it also accelerates the decay of load-bearing capacity. The reduction in deformation capacity can be attributed to the fact that greater axial pressure imposes more constraints on the specimen, leading to increased bond friction.

Figure 12b presents the skeleton curves for specimens C12B10, C10B10, and C8B10, which exhibit similar development trends. In the elastic stage, the initial stiffness of these three specimens is comparable, as it is primarily governed by the inner-core ordinary steel reinforcement when subjected to equal axial compression ratios. As the load increases, the skeleton curves decline relatively gradually after reaching the peak load. This behavior may be due to the secondary stiffness of the SFCB, which enables the structure to sustain a high load-carrying capacity even as damage occurs. Among these specimens, C12B10 exhibited the highest load capacity (50 kN), while C10B10 had the lowest, indicating that increased stiffness in the column longitudinal reinforcement effectively enhances the load capacity of the specimens.

Figure 12c illustrates the skeleton curves for specimens C8B6, C8B8, and C8B10. While the three curves are close during the elastic stage, noticeable differences emerge in the plastic stage, indicating that the fiber volume ratio of the beam longitudinal reinforcement significantly influences the seismic performance of the specimens. The load-carrying capacities of C8B8 and C8B10 are similar, whereas C8B6 demonstrates a lower load capacity. This reduction can be attributed to the smaller diameter of the inner core reinforcement in the SFCB, which hampers effective crack control, resulting in coarser cracks and more severe damage, ultimately reducing the specimen’s bearing capacity.

### 3.4. Energy Consumption

**Figure 13:**
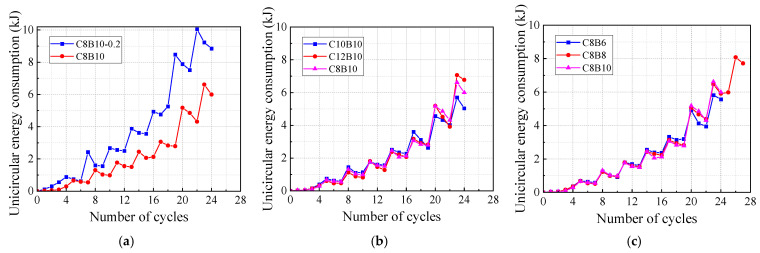
Unicircular energy consumption of specimens: (**a**) axial compression ratio group specimens; (**b**) column longitudinal reinforcement fiber volume rate group specimens; (**c**) beam longitudinal reinforcement fiber volume rate group specimens.

The energy consumption capacity of the specimens is analyzed through unicircular dissipation, cumulative dissipation, and equivalent viscous damping coefficient. The circumferential energy consumption of the specimen, represented by the area enclosed within a single hysteresis curve, is illustrated in Figure 13. The data indicates that increasing the axial compression ratio significantly enhances the unicircular energy consumption of the specimen, particularly in the later loading stages. Specifically, at the 23rd cycle (corresponding to an interlaminar displacement angle of 1/20), the axial compression ratio of 0.2 resulted in a 52% increase in energy consumption compared to a ratio of 0.1. Furthermore, among the specimens in the column SFCB longitudinal fiber volume rate group, the differences in unicircular energy consumption were minimal, with only slight variations observed in the number of loading cycles before damage occurred. At the 23rd cycle, specimen C12B10 exhibited a 24% increase in unicircular energy consumption relative to specimen C10B10, while specimen C8B10 showed a 16% increase. In the beam SFCB longitudinal bars fiber volume rate group, the unicircular energy consumption for the three specimens was comparable during the first 16 cycles. This can be attributed to the larger fiber volume rate in the SFCB longitudinal bars of specimen C8B6, as well as the elasticity of the outsourced FRP material, which contributes to strong deformation capacity and enhances energy consumption. However, in the later stages of loading, excessive concrete deformation led to a reduction in both the load-carrying capacity and energy consumption capacity of specimen C8B6. In contrast, specimen C8B8 demonstrated superior deformation and energy consumption capacities compared to both C8B6 and C8B10, owing to its relatively higher secondary and initial stiffness.

**Figure 14:**
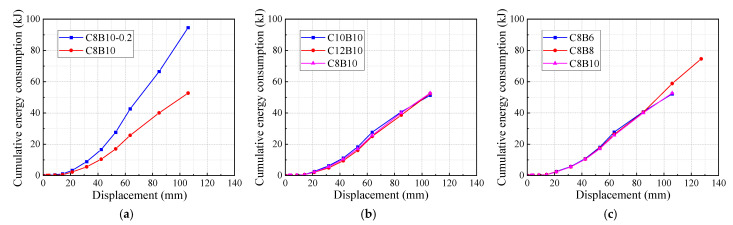
Cumulative energy consumption of specimens: (**a**) axial compression ratio group specimens; (**b**) column longitudinal reinforcement fiber volume rate group specimens; (**c**) beam longitudinal reinforcement fiber volume rate group specimens.

Cumulative energy consumption reflects the total energy absorbed by the specimen throughout the static test and is calculated by summing the energy dissipated during each loading cycle. The cumulative energy consumption of the specimens is illustrated in Figure 14. Comparative analysis reveals that the axial compression ratio significantly influences cumulative energy consumption; specimens with an axial compression ratio of 0.2 exhibited much higher cumulative energy consumption than those with a ratio of 0.1. Notably, at the point of damage, the cumulative energy consumption of specimen C8B10-0.2 was 80% greater than that of specimen C8B10. Additionally, the cumulative energy consumption among specimens in the column SFCB longitudinal fiber volume rate group and the beam SFCB longitudinal fiber volume rate group was similar. However, specimen C12B10 demonstrated slightly higher cumulative energy consumption compared to C10B10, while specimen C8B8 significantly outperformed specimens C8B6 and C8B10, with cumulative energy consumption values that were 43% and 41.6% higher, respectively, due to its ability to sustain larger displacements.

**Figure 15:**
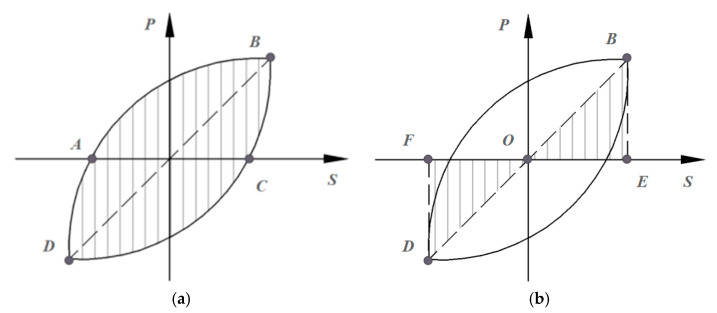
Diagram of equivalent viscous coefficient and energy consumption coefficient: (**a**) *S_ABCD_*; (**b**) *S_OBE_* and *S_ODF_*.

According to the JGJ/T101-2015 [43], the calculation schematic for the equivalent viscous damping coefficient and energy consumption coefficient is illustrated in Figure 15. The formulas for calculating the energy consumption coefficient and the equivalent viscous damping coefficient are provided in Equations (1) and (2).

(1) $$E=\frac{S_{ABCD}}{S_{OBE}+S_{ODF}}$$

(2) $$\xi_{\mathrm{eq}}=\frac{1}{2\pi }\frac{S_{ABCD}}{S_{OBE}+S_{ODF}}$$

**Figure 16:**
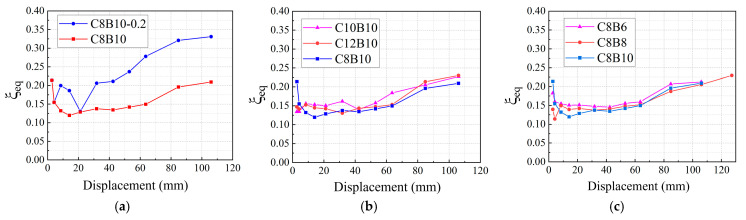
Equivalent viscous damping coefficient of the specimen: (**a**) axial compression ratio group specimens; (**b**) column longitudinal reinforcement fiber volume rate group specimens; (**c**) B = beam longitudinal reinforcement fiber volume rate group specimens.

As shown in Figure 16, the specimens continued to exhibit a certain growth trend even after damage. Notably, the growth slopes of specimens C8B8 and C12B10 were steeper at the time of damage, suggesting that the SFCB specimens retained good energy consumption capacity even after damage. This behavior highlights the contribution of the secondary stiffness of the SFCB, which enhances the energy consumption capacity of the specimens. Similarly, the axial compression ratio significantly affected the equivalent viscous damping coefficient of the joints. Increasing the axial compression ratio substantially improved the equivalent viscous damping coefficient. On the other hand, the fiber volumetric ratio of the column SFCB longitudinal reinforcement and the beam SFCB longitudinal reinforcement had little impact on the equivalent viscous damping coefficient. Specimens C10B10 and C8B6 exhibited slightly higher equivalent viscous damping coefficients in the intermediate section, which may be attributed to the larger cracks that developed in the concrete during the intermediate loading stage, including diagonal cracks in the core region. However, as the loading displacement increased, the concrete in these specimens experienced excessive deformation and damage, which slowed the growth of their viscous damping coefficients. Eventually, the equivalent viscous damping coefficients of these specimens became equal to or slightly lower than those of the other specimens in the same group.

### 3.5. Fixed-End Rotations of Column and Beam

**Figure 17:**
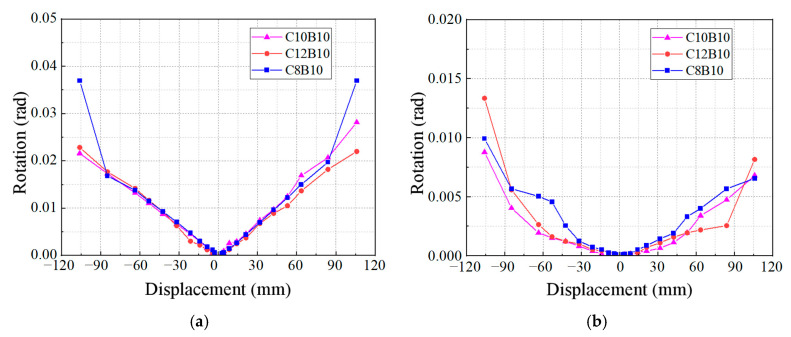
Effect of longitudinal reinforcement fiber volume rate in columns: (**a**) beam end corners; (**b**) column end corners.

The effects of the column longitudinal reinforcement fiber volume rates on the beam and column end angles of the specimens are illustrated in Figure 17. From the figure, it is evident that the three longitudinal fiber volume rates have minimal influence on the beam end angle of the specimens. The relationship between the beam end angle and the displacement for all three specimens appears to be approximately linear. In contrast, the column end angle is affected by the “off-axis” phenomenon during the loading process, resulting in asymmetrical column end angles at the same positive and negative loading displacements. Additionally, as the fiber volume rate of the longitudinal bars increases—leading to a decrease in longitudinal bar stiffness—the column end angle of the specimen at the same loading displacement tends to increase.

**Figure 18:**
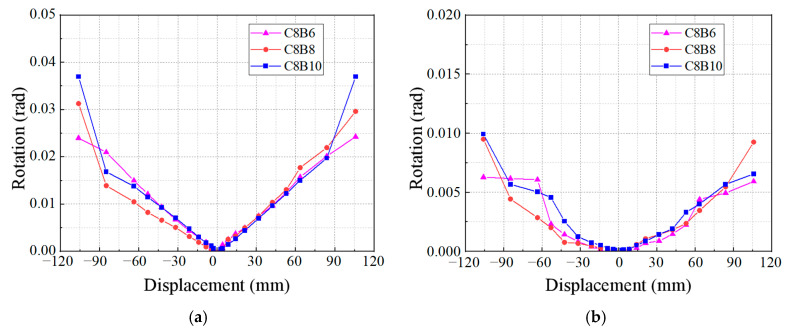
Effect of longitudinal reinforcement fiber volume rate in beams: (**a**) beam end corners; (**b**) column end corners.

Figure 18 depicts the effect of the beam longitudinal reinforcement fiber volume rate on the beam and column end turn angles of the specimens. In the final loading stage (3/50), the beam end turn angle of the specimens decreases as the beam longitudinal reinforcement fiber volume rate increases. This phenomenon may be attributed to two main factors: first, an increase in the longitudinal reinforcement fiber volume rate enhances the stiffness of the tendons in the beam, allowing it to resist a larger bending moment, which typically would result in an increase in the beam end turn angle. However, second, when the longitudinal bar fiber volume rate is increased, the exterior fibers experience greater forces after the reinforcement yields, which in turn reduces the beam end turn angle at the joint. The column end turn angle of the specimens exhibited a nonlinear relationship with displacement, with specimen C8B6 demonstrating a smaller column end turn angle compared to the others.

### 3.6. SFCB Strains

**Figure 19:**
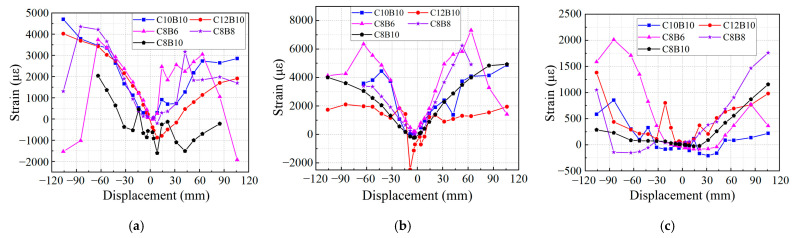
Strain–displacement curves of longitudinal bars: (**a**) beam longitudinal bar L3 position; (**b**) beam longitudinal bar L5 position; (**c**) column longitudinal bar Z2 position.

**Figure 20:**
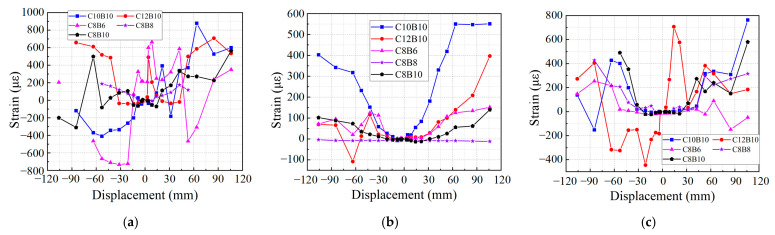
Strain–displacement curves of stirrups: (**a**) beam hoop LG1 position; (**b**) column hoop G1 position; (**c**) column hoop G5 position.

Due to the presence of numerous strain gauge measurement points and the premature failure of some strain gauges during the loading process, this study selects the most significant locations for strain measurements, prioritizing those where the strain gauges remained intact throughout loading. The longitudinal tendon strain curves of the beam are presented in Figure 19a,b. The data indicate that the longitudinal bar strains of all specimens exceeded 2200, signifying that the core reinforcement of the SFCB bars reached yielding, with the reinforcement primarily in a tensile state during the late loading stages. Notably, the beam longitudinal bar strain of specimen C12B10 is relatively small, while specimen C8B6 exhibits a larger strain. This discrepancy suggests that concrete cracks in specimen C8B6 developed more extensively, causing upward growth of the tensile cracks and resulting in increased tensile height, which in turn elevated the longitudinal tendon strains in this specimen. Additionally, the strain at the position of column longitudinal bar Z2 is depicted in Figure 19c. The results show that the strains of the column longitudinal tendons did not reach the yield strain of the reinforcement, indicating that the column cross-section remained relatively intact during the loading process. This observation aligns with the design specification’s requirement for “strong columns and weak beams”. During the push–pull loading, the longitudinal bars of all specimens were in tension. The strain curves of the hoop bars are illustrated in Figure 20. The strains of all the hoops are relatively low, with the strains of the beam hoops, column hoops, and joint core area hoops remaining below 800, significantly smaller than those of the beam longitudinal tendons. This finding corresponds with the observation that all specimens experienced damage at the beam end, characterized by small and fine diagonal cracks.

## 4. Conclusions

This study addresses the critical gap in understanding the seismic behavior of SFCB-reinforced seawater sea–sand concrete beam-–column joints, which has hindered their marine applications. Key findings include the following:

(1) All specimens showed beam end bending failure, in line with the design principle of ‘strong column and weak beam’. The longitudinal reinforcement of the column section did not reach yield strain, and the stirrup strain in the core area was low (<800 µε), indicating no significant shear failure occurred in the core area of the joint. Additionally, the combination of SFCBs and seawater sand concrete exhibits excellent bonding properties and crack resistance in a corrosive environment, with no sliding failure of the FRP bars.
(2) Increasing the axial compression ratio (e.g., from 0.1 to 0.2) significantly enhances the shear and bond strength of the joints, effectively inhibiting crack propagation and slip, but also accelerating bearing capacity degradation. At the same displacement angle, the energy consumption of the specimen with an axial compression ratio of 0.2 (e.g., C8B10-0.2) is 52% higher than that of the specimen with 0.1, and the cumulative energy consumption is 80% higher.
(3) An increase in the longitudinal SFCB fiber volume ratio (e.g., from 64% to 84%) improves joint stiffness, fracture distribution uniformity, and ductility. The C12B10 specimen, with the highest fiber volume ratio, exhibited the highest bearing capacity (50 kN), 16.7% and 8% higher than C8B10 and C10B10, respectively.
(4) The longitudinal SFCB fiber volume ratio significantly affects the seismic performance of the beam. The specimen with a medium fiber volume ratio (75%) (e.g., C8B8) demonstrated the best performance, with cumulative energy consumption 43% and 41.6% higher than C8B6 and C8B10, respectively. Its cracks were finer, and the concrete integrity was better. A low volume ratio (e.g., 60.9%) resulted in significant cracking of the concrete and a decrease in bearing capacity, while a high volume ratio (e.g., 86%) may reduce energy dissipation efficiency due to stiffness imbalance.
(5) The stable secondary stiffness of SFCBs provides continuous bearing capacity during the joint plastic stage, delays stiffness degradation, and improves the equivalent viscous damping coefficient of the specimen. For instance, C8B8 still maintains high energy dissipation capacity after failure.

## Figures and Tables

**Table 1:** Mix proportion of seawater sand concrete.

Cement (kg/m^3^)	Sand (kg/m^3^)	Stone (kg/m^3^)	Fly Ash (kg/m^3^)	Mineral Powder (kg/m^3^)	Seawater (kg/m^3^)	Water Reducer (kg/m^3^)
252	779	1034	54	54	170	7.96

**Table 2:** Physical properties of crushed stone.

Particle Size (mm)	Flakiness and Elongation Index (%)	Apparent Density (kg/m^3^)	Bulk Density (kg/m^3^)	Fines Content (%)
5~25	4	2620	1510	0.40

**Table 3:** Artificial seawater composition mix ratio.

Component	Concentration (g/L)
NaCl	24.530
MgCl_2_	5.200
Na_2_SO_4_	4.090
CaCl_2_	1.160
KCl	0.695
NaHCO_3_	0.201
KBr	0.101
H_3_BO_3_	0.027
SrCl_2_	0.025
NaF	0.003

**Table 4:** Reinforcement tensile specimen size.

Specimens	Diameter d (mm)	Anchor Section Length *l*_1_ (mm)	Working Section Length *l*_0_ (mm)	Overall Length *l* (mm)
SFCB	16	200	350	750
SFCB	20	300	400	1000
GFRP	10	150	200	500

**Table 5:** Mechanical properties of reinforcement.

Specimens ^1^	Diameter d (mm)	Modulus of Elasticity *E_s_* (GPa)	Secondary Rigidity *E_s_^′^* (GPa)	Yielding Strength *f*_y_ (MPa)	Ultimate Strength *f_u_* (MPa)
S6	6	206.21	/	559.28	759.31
S8	8	186.86	/	525.36	714.63
S10	10	202.33	/	523.73	649.38
S12	12	205.72	/	518.17	622.66
G10	10	44.75	/	/	770.82
S6G16	16	71.35	35.81	177.48	771.28
S8G16	16	84.22	32.19	211.76	746.75
S10G16	16	104.69	31.01	233.73	722.28
S8G20	20	64.92	34.71	189.06	811.66
S10G20	20	72.87	32.97	181.05	793.27
S12G20	20	90.15	22.39	235.23	785.68

^1^ “S” represents steel, “G” denotes GFRP, and “S6G16” refers to an SFCB with an inner-core steel bar of 6 mm and an outer diameter of 16 mm. Other specimens are named similarly.

**Table 6:** Specimen design parameters.

Specimens	Column Longitudinal Bar			Beam Longitudinal Bar			Axial Compression Ratio
	Configuration	RR ^1^ (%)	FVR ^1^ (%)	Configuration	RR (%)	FVR (%)	
C8B10	6S8G20	2.09	84	4S10G16	1.58	60.9	0.1
C8B10-0.2	6S8G20	2.09	84	4S10G16	1.58	60.9	0.2
C10B10	6S10G20	2.09	75	4S10G16	1.58	60.9	0.1
C12B10	6S12G20	2.09	64	4S10G16	1.58	60.9	0.1
C8B6	6S8G20	2.09	84	4S6G16	1.58	86	0.1
C8B8	6S8G20	2.09	84	4S8G16	1.58	75	0.1
C8B10	6S8G20	2.09	84	4S10G16	1.58	60.9	0.1
C8B10-0.2	6S8G20	2.09	84	4S10G16	1.58	60.9	0.2

^1^ RR-Reinforcement ratio, FVR-Fiber volume ratio, in which fibers and resin are considered together.

**Table 7:** Specimen ductility ratio.

Specimens	P*_y_* (kN)	Δ*_y_* (mm)	P*_m_* (kN)	Δ*_m_* (mm)	P*_u_* (kN)	Δ*_u_* (mm)	DC	Average Value
C8B10-0.2	45.80	35.80	53.94	84.52	45.80	105.95	2.96	2.88
	−44.13	−37.92	−54.70	−85.09	−49.82	−106.2	2.80	
C10B10	37.19	28.80	43.23	45.30	36.46	88.28	3.07	3.31
	−42.36	−29.90	−49.23	−63.70	−46.21	−106.10	3.55	
C12B10	45.36	35.61	50.46	67.05	47.66	105.95	2.98	2.79
	−41.00	−40.64	−47.81	−67.25	−44.68	−106.05	2.61	
C8B6	39.13	40.07	44.24	72.62	37.60	103.64	2.59	2.62
	−44.13	−40.14	−53.48	−63.88	−45.71	−106.28	2.65	
C8B8	39.10	33.02	46.38	65.44	40.28	127.14	3.85	3.54
	−49.81	−39.49	−56.06	−87.26	−49.51	−127.26	3.22	
C8B10	41.53	33.05	46.50	65.97	41.58	108.77	3.29	3.26
	−50.11	−35.17	−53.78	−88.64	−49.94	−113.54	3.23	

## Data Availability

The original contributions presented in this study are included in the article. Further inquiries can be directed to the corresponding author.
